# Exact solitary wave propagations for the stochastic Burgers’ equation under the influence of white noise and its comparison with computational scheme

**DOI:** 10.1038/s41598-024-58553-2

**Published:** 2024-05-09

**Authors:** Muhammad Zafarullah Baber, Wael W. Mohammed, Nauman Ahmed, Muhammad Sajid Iqbal

**Affiliations:** 1 Department of Mathematics and Statistics, The University of Lahore, Lahore, Pakistan; 2 Department of Mathematics, Faculty of Science, University of Ha’il, Ha’il, 2440 Saudi Arabia; 3 Department of Mathematics, Faculty of Science, Mansoura University, Mansoura, 35516 Egypt; 4 Department of Computer Science and Mathematics, Lebanese American University, Beirut, Lebanon; 5 Department of Academic Affairs, School of Leadership and Business, Oryx Universal College with Liverpool John Moores University (UK), 12253 Doha, Qatar; 6 Department of Humanities and Basic Science, MCS, National University of Science and Technology, Islamabad, Pakistan

**Keywords:** Stochastic Burgers’ equation, Proposed SFD scheme, Analysis of the scheme, Exact stochastic solutions, GERF method, Applied mathematics, Physics

## Abstract

In this manuscript, the well-known stochastic Burgers’ equation in under investigation numerically and analytically. The stochastic Burgers’ equation plays an important role in the fields of applied mathematics such as fluid dynamics, gas dynamics, traffic flow, and nonlinear acoustics. This study is presented the existence, approximate, and exact stochastic solitary wave results. The existence of results is shown by the help of Schauder fixed point theorem. For the approximate results the proposed stochastic finite difference scheme is constructed. The analysis of the proposed scheme is analyzed by presented the consistency and stability of scheme. The consistency is checked under the mean square sense while the stability condition is gained by the help of Von-Neumann criteria. Meanwhile, the stochastic exact solutions are constructed by using the generalized exponential rational function method. These exact stochastic solutions are obtained in the form of hyperbolic, trigonometric and exponential functions. Mainly, the comparison of both numerical and exact solutions are analyzed via simulations. The unique physical problems are constructed from the newly constructed soliton solutions to compare the numerical results with exact solutions under the presence of randomness. The 3D and line plots are dispatched that are shown the similar behavior by choosing the different values of parameters. These results are the main innovation of this study under the noise effects.

## Introduction

In recent years, physics, climatic dynamics, biology, economics, geophysics, and other subjects have made extensive use of the significance of randomness in modelling, analysing, complex media, and simulations. The differential equations (DEs) which are containing the random fluctuations under time are called stochastic (SDEs)^1,2^. The SDEs or stochastic partial differential equations (SPDEs) are suitable mathematical modeling for complex systems under multiplicative time noise. The SPDEs are very crucial to deal with numerically and analytically as well^3,4^. There are many mathematical techniques are developed to explore the SPDEs for the approximate solution and exact solution as well. Iqbal et al. proposed the stochastic forward Euler scheme and stochastic Crank–Nicolson scheme to investigate approximate solutions for nonlinear stochastic NWS equation^5^. The NSFD scheme is proposed by Arif et al. and computed the numerical results for stochastic Reaction–Diffusion Nonlinear Chemical Model^6^. Raza et al. also applied the NSFD scheme to investigated the stochastic dengue epidemic model^7^. Kovács proposed the backward Euler scheme to gained approximated results for the stochastic Allen–Cahn equation^8^. Recently, yasin et al. proposed the stochastic standard finite difference scheme^9^, stochastic forward Euler difference scheme^10^, NSFD scheme^11^ and analyzed these techniques are consistency or stable.

Finding the exact solutions of SPDEs are also a difficult task while some researchers are working on that. Mohammed et al. investigated the soliton solutions for the Fractional Stochastic Kraenkel–Manna–Merle Equations using the mapping approach^12^, by applying the F-expansion method he explored the solitary wave solutions^13^. He considered the stochastic shallow water wave equations and gained the soliton solutions via He’s iterational approch^14,15^. Albosaily et al. investigated the exact solutions for the fractional stochastic Fokas–Lenells equation by applying the modified mapping method^16^. Shaikh et al. applying the F-expansion method to investigated the stochastic Konno–Oono system under the noise effect^17^ and also investigated the solitary wave structures for the stochastic Nizhnik–Novikov–Veselov (SNNV) system^18^. Assiri et al. worked on the optical solitary waves solutions for the third order dispersive Schrödinger equation^19^ and Khan et al., worked on the optical soliton solutions for the new generalized nonlinear Schrödinger equation^20^. Ali et al., constructed the solitary wave solutions for the fractional Wazwaz–Benjamin–Bona–Mahony system^21^, Chahlaoui et al., analyzed the soliton solution, modulation instability and sensitive analysis to fractional nonlinear Schrödinger model with Kerr Law nonlinearity^22^ and Ali et al., constructed the exact soliton solutions and stability analysis for the (3+ 1)-dimensional nonlinear Schrödinger model^23^.

Burgers’ equation, a famous partial differential equation, is crucial to the study of applied mathematics in the areas of fluid dynamics, gas dynamics, traffic flow, and nonlinear acoustics, among others^24^. Although a force is given to the mathematical model, the deterministic Burgers’ equations do not perform any chaos, hence they cannot adequately explain turbulence. If the force is changed to one that is random, the outcomes are completely different. Otherwise, all deterministic Burgers’ equation solutions converge to distinct stationary spots as time increases indefinitely^25^. Due to the presence of certain additional forces, such as turbulent dynamics and instability, we must generalize Burgers’ equation by taking more factors into consideration. In the Ito sense, the stochastic Burgers’ equation with multiplicative noise is considered as follows^26^:

1 $$\begin{aligned} \Upsilon _{t} + \Upsilon \Upsilon _{x}=\nu \Upsilon _{xx}+\sigma \Upsilon \beta _t, \end{aligned}$$

where $\nu$ is positive constants and $\beta _t$ is the multiplicative time noise of standard Wiener process $\beta (t)$. There are many analysis are done on the classical Burgers’ equation but the stochastic version of the Burgers’ equation is investigate by some researchers analytically. Mohammed et al. investigated the exact solutions by using the $\exp (-\phi (\mu ))$-expansion method and $G'/G$-expansion method^25,26^. Numerically approach namely as Galerkin approximation is done by Blomker et al.^27^, Kutluay et al. find the numerical solutions for the one-dimensional Burgers equation by using the explicit and exact-explicit finite difference methods^28^. Xie et al. used reproducing kernel function to obtained the numerical solution of one-dimensional Burgers’ equation^29^.

The advantages of stochastic finite difference scheme are, such as it is easy to compute, it is time efficient, high efficient computers are required for implicit methods and for forward method low efficient computers can be used. While the GERF method is the technique that will provided us the Dark, Bright, combined and periodic function solutions. In this study, we investigate the stochastic Burgers equation under the effect of noise. The solutions to the SPDEs cannot be found in the classical theory of PDEs. The creation of a theory that explains the physical behavior of randomness in classical PDEs is urgently needed. For the existence of results under the Banach space, A-priori bounds are guaranteed. The Schauder fixed point theorem is used to demonstrate the existence results. Furthermore, we propose an NSFD scheme for the numerical results. It is important that the scheme is consistent and stable. So, we check the consistency of the scheme under the mean square sense while the stability condition is gained by Von-Neumann criteria. Moreover, the stochastic exact solitary wave solutions are extracted by using the GERM method. The motivation of this study is that we compare the numerical results with exact solitary wave solutions by unique physical problems. The numerical results are successfully compared with exact solitary wave solutions and almost they will give us the same results. These results are the main innovation of this study under the noise effects.

### Theorem 1

Wiener process and It${\hat{o}}$ integral: The Brownian motion $\beta (t)$ is said to be stochastic process $\{\beta (t)\}_{t\ge 0}$ if it satisfy the following properties^30^

1. $$\beta (0)=0$$ with probability 1.
2. $\beta (t)$ is the continuous function of $t\ge 0$.
3. $\beta (t_2)-\beta (t_1)$ and $\beta (t_4)-\beta (t_3)$ are independent increments for all $0\le t_1<t_2\le t_3<t_4$.
4. $\beta (t_2)-\beta (t_1)$, $\beta (t_4)-\beta (t_3)$ has normal distribution ${\mathfrak {N}}(0,(t_2-t_1),(t_4-t_3))$.
5. ${\mathbb {E}}\bigg [\beta _t\bigg ]=0$ for each value $t\ge 0$.
6. ${\mathbb {E}}\bigg [\beta _t^2\bigg ]=t$ for each value $t\ge 0$.
7. $${\mathbb {E}}\bigg [\beta (t)-\beta (s)\bigg ]=0$$.
8. $${\mathbb {E}}\bigg [(\beta (t)-\beta (s))^2\bigg ]=t-s$$.

### Lemma 1

It${\hat{o}}$ stochastic integral: The square property of an It${\hat{o}}$ integral is $\int _0^t \sigma _t d\beta (t)$ has the following property

2 $$\begin{aligned} {\textbf{E}} \bigg |\int \limits _0^t \sigma _t d\beta (t)\bigg |^2=\int \limits _0^t {\mathbb {E}}\bigg | \sigma _t\bigg |^2 dt. \end{aligned}$$

## Existence result

Here, we focus on the existence of result for the stochastic Burgers’ equation by applying the Schauder fixed point theorem. The Schauder fixed point theorem is stated as^31^;

### Theorem 2

Suppose $\Upsilon$ be continuous mapping a Banach space $\mathbb {C}$ and a closed convex subset ${\textbf{B}}$ have self mapping and if $\Upsilon ({\textbf{B}})$ is relatively compact. Then $\Upsilon$ has at one fixed point in ${\textbf{B}}$ .

So, we convert Eq. (1) into operator form by using the heat kernels and simply voltera integral equation as the inversion of the PDE Eq. (1) such as

3 $$\begin{aligned} \Upsilon (x,t)=\mathbf {\Upsilon }_\rho (x,t)=a_0+\int _0^t(\nu \Upsilon _{xx}(x, \rho )-\Upsilon (x, \rho )\Upsilon _x(x, \rho )+\sigma \Upsilon (x, \rho ){\dot{\beta }}(\rho ))d\rho , \end{aligned}$$

here we take $a_0$ as a integration constant usually that is taken as a initial condition. Equation (3) serve as a fixed point operator Eq. (1) and any fixed point of Eq. (3) is the solution not only for this Eq. (3) but also the solutions of original Eq. (1) as well. To, show the existence of result for the Eq. (1) we shall apply the fixe point theorem which is stated such as

### Theorem 3

Suppose a Ball ${\textbf{B}}$ in the Banach space $\mathbb {C}$ which is closed and convex subset of ${\mathbb {C}}$. Also, suppose a continuous function $\Upsilon$ that will be satisfy these two condition as follows

- $$\Upsilon : {\textbf{B}}\rightarrow {\textbf{B}}$$,
- $\Upsilon$ is relative compact,

thus $\Upsilon$ has at least of fixed point in Ball ${\textbf{B}}$.

Now, apply this result on Eq. (3), we suppose the space of continuous function with it suprimum norm and a Ball ${\textbf{B}}$ with is defined as

4 $$\begin{aligned} {\textbf{B}}(\varpi )=\{a_0, \quad a_0\in [0, \rho ], \quad \Vert \Upsilon \Vert \le \tau \}. \end{aligned}$$

For the first condition self mapping

5 $$\begin{aligned} \Upsilon : {\textbf{B}}(\varpi )\rightarrow {\textbf{B}}(\varpi ), \end{aligned}$$

$\Rightarrow$ Equation (3) is taking the form

6 $$\begin{aligned} \Vert \mathbf {\Upsilon }\Vert \le \Vert a_0\Vert +\int _0^t(\nu \Vert \Upsilon _{xx}\Vert +\Vert \Upsilon \Vert \Vert \Upsilon _x\Vert +\sigma \Vert \Upsilon \Vert \Vert {\dot{\beta }}(\rho )\Vert )d\rho , \end{aligned}$$

where $\Upsilon$ is positive function while $\Vert \Upsilon _{xx}\Vert \le \kappa _1$, $\Vert \Upsilon _{x}\Vert \le \kappa _2$ and we suppose that $\Vert {\dot{\beta }}(\rho )\Vert =\kappa _3$ is bounded noise.

7 $$\begin{aligned} \Vert \mathbf {\Upsilon }\Vert \le \alpha +\rho (\nu \kappa _1+\tau \kappa _2+\sigma \tau \kappa _3)\le \tau , \end{aligned}$$

8 $$\begin{aligned} \rho \le \frac{\tau -\alpha }{\nu \kappa _1+\tau \kappa _2+\sigma \tau \kappa _2}. \end{aligned}$$

For the second condition (Relative compactness) we consider the images $\mathbf {\Upsilon }_i$ and its pre-images $\Upsilon _i$ then fixed point is by specified $t^{*}$, we can easily verified that

9 $$\begin{aligned} \Vert \mathbf {\Upsilon }_i(t)-\Upsilon _i(t^*)\Vert \le H_\Upsilon |t-t^*|, \end{aligned}$$

where $H_\Upsilon$ is positive constant. While $\mathbf {\Upsilon }_i$ is equi-continues. Hence the Ball ${\mathbb {B}}_{\tau }(\varpi )$ is relatively compact. Hence by Schauder fixed point theorem at least one solution is exist in the interval,

10 $$\begin{aligned} \bigg [0, \min \left( \frac{\tau -\alpha }{\nu \kappa _1+\tau \kappa _2+\sigma \tau \kappa _2}\right) \bigg ]. \end{aligned}$$

**Figure 1:**
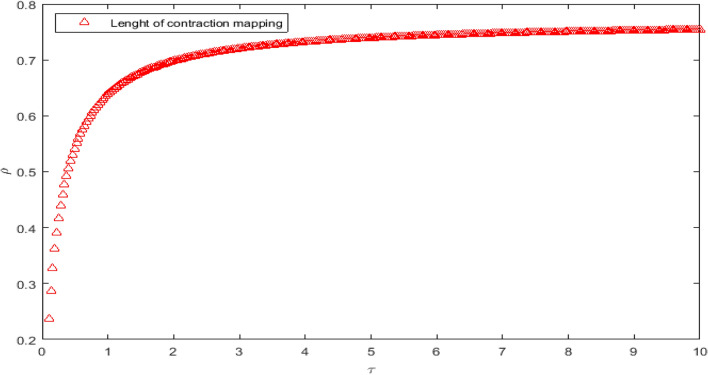
Length of continuity for $\rho$.

The Fig. 1 id plot for the condition $\rho$ which is gain for the length of contraction in Eq. (8) using the parameters values such as $\alpha =0.01, \nu =0.5, \kappa _1=0.5, \kappa _2=2, \kappa _3=0.3, \sigma =0.2.$ This figure show the length on the contraction mapping which show the bound in interval $C\in [0, \rho ]$.

## Proposed SFDS

This current sections, is deals with the propose finite difference scheme (SFDS). We approximate the continuous function $\Upsilon (x,t)$ with approximate solutions $\Upsilon _c^d$ and its derivatives such as

11 $$\begin{aligned} \Upsilon _t \approx \frac{\Upsilon _c^{d+1}-\Upsilon _c^d}{k}, \qquad \Upsilon _x \approx \frac{\Upsilon _{c+1}^{d}-\Upsilon _c^d}{h}, \qquad \Upsilon _{xx}\approx \frac{\Upsilon _{c+1}^d-2 \Upsilon _c^{d}+\Upsilon _{c-1}^d}{h^2}, \end{aligned}$$

here we suppose $k=\Delta t$ and $h=\Delta x$ that are the are time and space stepsizes respectively. Substituting these values in the Eq. (1) and obtained the SFDS as follows

12 $$\begin{aligned} \Upsilon _c^{d+1}=\alpha \bigg (\Upsilon _{c-1}^d+\Upsilon _{c+1}^d\bigg )+(1-2\alpha ) \Upsilon _c^d+\gamma \bigg (\Upsilon _c^d\bigg )^2 -\gamma \Upsilon _c^d \Upsilon _{c+1}^d+\sigma \Upsilon _c^d \bigg (\beta ^{(d+1)k}-\beta ^{dk}\bigg ), \end{aligned}$$

where $\gamma =\frac{\Delta t}{\Delta x}$ and $\alpha =\frac{\nu \Delta t}{\Delta x^2}$. So, this is the required SFDS for the Eq. (1).

## Consistency of scheme

This current section, deals with the consistency of the proposed finite different scheme, which is proved by the help of mean square sense^32,33^.

### Theorem 4

The proposed SFDS is consistency for $\Upsilon$ in Eq. (12) is shows the consistency with the Eq. (1) in the mean square sense.

### Proof

We take a operator ${\mathbb {B}}(\Upsilon )=\int _{d\Delta t}^{(d+1)\Delta t}(\Upsilon ) ds$ for the smooth function $\Upsilon (x, t)$. So, apply this operator on Eq. (1) and get the expression as follows

13 $$\begin{aligned} {\mathbb {B}}(\Upsilon )_c^d{} & {} =\Upsilon (c\Delta x, (d+1)\Delta t)-\Upsilon (c\Delta x, d\Delta t)+\int _{d\Delta t}^{(d+1)\Delta t}\Upsilon (d\Delta x, s)\Upsilon _x(d\Delta x, s) ds\nonumber \\{} & {} \quad -\nu \int _{d\Delta t}^{(d+1)\Delta t} \Upsilon _{xx}(d\Delta x, s) ds-\sigma \int _{d\Delta t}^{(d+1)\Delta t} \Upsilon (d\Delta x, s) d \beta |_s, \end{aligned}$$

14 $$\begin{aligned} {\mathbb {B}}|_c^d(\Upsilon ){} & {} =\Upsilon (c\Delta x, (d+1)\Delta t)-\Upsilon (c\Delta x, d\Delta t)+ \frac{\Delta t}{\Delta x} \Upsilon (c\Delta x, d \Delta t)\left( \Upsilon ((c+1)\Delta x, d\Delta t)\right. \nonumber \\{} & {} \left. \quad -\Upsilon (c\Delta x, d \Delta t)\right) -\frac{\nu }{\Delta x^2}(\Upsilon ((c+1)\Delta x,d \Delta t)-2 \Upsilon (c\Delta x, (d+1)\Delta t) \nonumber \\{} & {} \quad +\Upsilon ((c-1)\Delta x, d\Delta t))+ \sigma \Upsilon (c\Delta t, d\Delta x) (\beta ^{(d+1)\Delta t}-\beta ^{d\Delta }), \end{aligned}$$

the above relations can be written by the mean square sense as follows

15 $$\begin{aligned} {\mathbb {E}}\bigg |{\mathbb {B}}(\Upsilon )_c^d-{\mathbb {B}}|_c^d(\Upsilon )\bigg |^2{} & {} \le {\mathbb {E}}\bigg |\int _{d\Delta t}^{(d+1)\Delta t}\Upsilon (d\Delta x, s)\Upsilon _x(d\Delta x, s)- \frac{\Delta t}{\Delta x} \Upsilon (c\Delta x, d \Delta t)\left( \Upsilon ((c+1)\Delta x, d\Delta t)\right. \nonumber \\{} & {} \left. \quad -\Upsilon (c\Delta x, d \Delta t)\right) ds\bigg |^2 -4 {\mathbb {E}}\nu ^2 \bigg |\int _{d\Delta t}^{(d+1)\Delta t} \Upsilon _{xx}(d\Delta x, s)+\frac{1}{\Delta x^2}(\Upsilon ((c+1)\Delta x,d \Delta t)\nonumber \\{} & {} \quad -2 \Upsilon (c\Delta x, (d+1)\Delta t)ds\bigg |^2-4 \sigma ^2 {\mathbb {E}}\bigg |\int _{d\Delta t}^{(d+1)\Delta t}( \Upsilon (d\Delta x, s)-\Upsilon (c\Delta t, d\Delta x)) d \beta |_s\bigg |^2, \end{aligned}$$

using the property of Ito integral we obtain

16 $$\begin{aligned} {\mathbb {E}}\bigg |{\mathbb {B}}(\Upsilon )_c^d-{\mathbb {B}}|_c^d(\Upsilon )\bigg |^2{} & {} \le \int _{d\Delta t}^{(d+1)\Delta t}{\mathbb {E}}\bigg |\Upsilon (d\Delta x, s)\Upsilon _x(d\Delta x, s)- \frac{\Delta t}{\Delta x} \Upsilon (c\Delta x, d \Delta t)\left( \Upsilon ((c+1)\Delta x, d\Delta t)\right. \nonumber \\{} & {} \left. \quad -\Upsilon (c\Delta x, d \Delta t)\right) ds\bigg |^2 -4 \nu ^2 \int _{d\Delta t}^{(d+1)\Delta t} {\mathbb {E}}\bigg |\Upsilon _{xx}(d\Delta x, s)-\frac{1}{\Delta x^2}(\Upsilon ((c+1)\Delta x,d \Delta t)\nonumber \\{} & {} \quad -2 \Upsilon (c\Delta x, (d+1)\Delta t)ds\bigg |^2-4 \sigma ^2 \int _{d\Delta t}^{(d+1)\Delta t}{\mathbb {E}}\bigg |( \Upsilon (d\Delta x, s)-\Upsilon (c\Delta t, d\Delta x)) ds\bigg |^2. \end{aligned}$$

$\bigg |{\mathbb {B}}(\Upsilon )_c^d-{\mathbb {B}}|_c^d(\Upsilon )\bigg |\rightarrow 0$ as $c\rightarrow \infty$, $d\rightarrow \infty$. Hence proposed scheme is consistent with the Eq. (1). $\square$

## Stability of scheme

This current section, deals with the Linearized stability analysis of the proposed finite different scheme^32–34^. The differential equation is replaced by $\Upsilon _{c, d}$ as

17 $$\begin{aligned} \Upsilon _{c, d}=G(t) e^{i(\rho x)}, \end{aligned}$$

substituting this into the scheme and obtained the amplification factor as

18 $$\begin{aligned} {\mathbb {E}}\le 1+\delta \Delta t, \end{aligned}$$

here $\delta$ is taken a constant. Hence this is the necessary condition for stability of our proposed scheme.

### Theorem 5

The proposed SFDS (12) is unconditionally stable with $(d+1)\Delta t+T$.

### Proof

The Von-Neumann criteria it applying to prove the stability of the proposed SFD scheme. So, we linearized the Eq. (12) such as

$$\begin{aligned} \Upsilon _c^{d+1}=\alpha \bigg (\Upsilon _{c-1}^d+\Upsilon _{c+1}^d\bigg )+(1-2\alpha )\Upsilon _c^d+\sigma \Upsilon _c^d \bigg (\beta ^{(d+1)k}-\beta ^{dk}\bigg ), \end{aligned}$$

putting the Eq. (17) in the above expression and obtain

$$\begin{aligned} G(t+\Delta t)e^{i\rho x}=\left( \alpha \bigg (e^{-i\rho \Delta x}+e^{i\rho \Delta x}\bigg )+(1-2\alpha )+\sigma \bigg (\beta ^{(d+1)k}-\beta ^{dk}\bigg )\right) G(t)e^{i\rho x}, \end{aligned}$$

19 $$\begin{aligned} {\mathbb {E}} \bigg |\frac{G(t+\Delta t)}{G(t)}\bigg |^2=\bigg |1-2\alpha +2\alpha \bigg (1-2\sin ^2\bigg (\frac{\rho \Delta x}{2}\bigg )\bigg )\bigg |^2+\bigg |\sigma \bigg |^2\bigg (\beta ^{(d+1)k}-\beta ^{dk}\bigg ), \end{aligned}$$

here, $\Upsilon$ is the independent from the state of Wiener process so we obtain

20 $$\begin{aligned} {\mathbb {E}} \bigg |\frac{G(t+\Delta t)}{G(t)}\bigg |^2=\bigg |1-2\alpha +2\alpha (1-2\sin ^2(\frac{\rho \Delta x}{2}))\bigg |^2+|\sigma |^2\Delta t, \end{aligned}$$

21 $$\begin{aligned} {\mathbb {E}} \bigg |\frac{G(t+\Delta t)}{G(t)}\bigg |^2=\bigg |1-4\sin ^2(\frac{\rho \Delta x}{2}))\bigg |^2+|\sigma |^2\Delta t, \end{aligned}$$

hence, $1-4\sin ^2\bigg (\frac{\rho \Delta x}{2}\bigg )\le 1$ and $\sigma =\delta$ then we obtain

22 $$\begin{aligned} {\mathbb {E}} \bigg |\frac{G(t+\Delta t)}{G(t)}\bigg |^2\le 1+\delta \Delta t. \end{aligned}$$

Hence the proposed scheme is unconditionally stable. $\square$

## Stochastic exact solutions

This current section, deals with the stochastic exact solitary wave (ESW) solutions for the stochastic Burgers’ equation. We applying the wave transformation such as^35,36^

23 $$\begin{aligned} \Upsilon (x, t)=\Theta (\rho )e^{\sigma \beta (t)-\frac{\sigma ^2}{2}t}, \qquad \text{ where } \qquad \rho =l x- c t, \end{aligned}$$

where $\Theta$ is deterministic function, *l* is amplitude of wave, *c* is the speed of light and $\sigma$ is the noise strength. The derivatives are taking as follows

$$\begin{aligned} \frac{d \Upsilon }{dt}= & {} \left( -c \Theta '+\sigma \Theta \beta _t-\frac{\sigma ^2}{2}\Theta +\frac{\sigma ^2}{2}\Theta \right) e^{\sigma \beta (t)-\frac{\sigma ^2}{2}t},\\ \frac{d \Upsilon }{dt}= & {} l \Theta 'e^{\sigma \beta (t)-\frac{\sigma ^2}{2}t},\\ \frac{d^2 \Upsilon }{dt^2}= & {} l^2 \Theta ''e^{\sigma \beta (t)-\frac{\sigma ^2}{2}t}, \end{aligned}$$

where $\frac{\sigma ^2}{2}\Theta (\rho )$ is referred to the It${\hat{o}}$ term. Substituting these values into the Eq. (1) and get

24 $$\begin{aligned} -c \Theta '+l \Theta \Theta 'e^{\sigma \beta (t)-\frac{\sigma ^2}{2}t}-\nu l^2\Theta ''=0. \end{aligned}$$

Now, we take the expectation on Eq. (24) such as

25 $$\begin{aligned} -c \Theta '+l \Theta \Theta '{\mathbb {E}}(e^{\sigma \beta (t)})e^{-\frac{\sigma ^2}{2}t}-\nu l^2\Theta ''=0. \end{aligned}$$

Therefore ${\mathbb {E}}(e^{\delta Z})$ for every $\delta$ is real number and *Z* is the standard normal random variable, the identity ${\mathbb {E}}(e^{\sigma \beta (t)})=e^{\frac{\sigma ^2}{2}t}$. So, Eq. (25) takes the form

26 $$\begin{aligned} -c \Theta '+l \Theta \Theta '-\nu l^2\Theta ''=0, \end{aligned}$$

where $\Theta$ is a polynomial and $'=\frac{d}{d\rho }$. To, reduce the order of the Eq. (27) integrate it once and get

27 $$\begin{aligned} -c \Theta +\frac{l}{2} \Theta ^2-\nu l^2\Theta '(\rho )=0. \end{aligned}$$

## GERF method

The generalize exponential rational function (GERF) method^37,38^ is use to construct the exact stochastic solutions for the stochastic Burgers’ equation. The general solution of the Eq. (27) is taken in the following form

28 $$\begin{aligned} \Theta (\rho )= a_0+\sum _{k=1}^N a_{k}J(\rho )^{k}+\sum _{k=1}^Nb_{k} J(\rho )^{-k}, \end{aligned}$$

where $a_{0}$, $a_{k}$ and $b_{k}$ are the constants, and

29 $$\begin{aligned} J(\rho )=\frac{A_1 e^{B_1\rho }+A_2 e^{B_2\rho }}{A_1 e^{B_1\rho }+A_2 e^{B_2\rho }}. \end{aligned}$$

To, obtained the value of *N* we applying the homogeneous balancing principle on highest derivative $\Theta ^{'}$ and nonlinear term $\Theta ^2$ and get $N=1$. Putting this value into (28) it can be expressed in the following polynomial.

30 $$\begin{aligned} \Theta (\rho )=a_0+a_1 J (\rho )+\frac{b_1}{J(\rho )}. \end{aligned}$$

### Family of solution 1

If we choose $[A_1, A_2, A_3,A_4]=[-1,-1,1,-1]$ and $[B_1,B_2,B_3,B_4]=[1,-1,1,-1]$, then Eq. (29) changes into,

31 $$\begin{aligned} \Lambda (\eta )=-\frac{\cosh (\rho )}{\sinh (\rho )}. \end{aligned}$$

Substituting Eq. (30) with the help of Eq. (31) into the Eq. (27), the set of equations is obtained. Using Mathematica to solve this set of equations will yield the following unknown constants such as:

- *Set 1* The unknown constants are $a_0=2 l \nu ,a_1=2 l \nu ,b_1=0 , c=2 l^2 \nu ,$, the singular stochastic soliton solution is obtained for Eq. (1) such as,
  32 $$\begin{aligned} \Upsilon _{1}(x,t)=\left( 2 l \nu -2 l \nu \coth \left( l x-2 l^2 \nu t\right) \right) e^{\sigma \beta (t)-\frac{\sigma ^2}{2}t}. \end{aligned}$$
- *Set 2* The unknown constants are $a_0=2 l \nu ,a_1=0,b_1=2 l \nu ,c=2 l^2 \nu$, the dark stochastic soliton solution is obtained for Eq. (1) such as,
  33 $$\begin{aligned} \Upsilon _{2}(x,t)=\left( 2 l \nu -2 l \nu \tanh \left( l x-2 l^2 \nu t\right) \right) e^{\sigma \beta (t)-\frac{\sigma ^2}{2}t}. \end{aligned}$$

### Family of solution 2

If $[A_1, A_2, A_3,A_4]= [-i, i, 1, 1]$, $[B_1,B_2,B_3,B_4]= [i, -i, i, -i]$, then Eq. (29) changes into,

34 $$\begin{aligned} \Lambda (\rho )=-\frac{\sin (\rho )}{\cos (\rho )}. \end{aligned}$$

Substituting Eq. (30) with the help of Eq. (34) into the Eq. (27) the set of equations is obtained. Using Mathematica to solve this set of equations will yield the following unknown constants such as:

- *Set 1* The unknown constants are $a_0=2 i l \nu ,a_1=2 l \nu ,b_1=0,c=2 i l^2 \nu ,$ the stochastic SWS of Eq. (1) is obtained as,
  35 $$\begin{aligned} \Upsilon _{3}(x,t)=\left( 2 i l \nu -2 l \nu \tan \left( l x-2 i l^2 \nu t\right) \right) e^{\sigma \beta (t)-\frac{\sigma ^2}{2}t}. \end{aligned}$$
- *Set 2* The unknown constants are $a_0=-4 i l \nu ,a_1=2 l \nu ,b_1=-2 l \nu ,c=-4 i l^2 \nu ,$ the stochastic SWS is obtained for Eq. (1) such as,
  36 $$\begin{aligned} \Upsilon _{4}(x,t)=\left( -2 l \nu \tan \left( l x+4 i l^2 \nu t\right) +2 l \nu \cot \left( l x+4 i l^2 \nu t\right) -4 i l \nu \right) e^{\sigma \beta (t)-\frac{\sigma ^2}{2}t}. \end{aligned}$$

### Family of solution 3

If $[A_1, A_2, A_3,A_4]=[1, 0, 1, 1]$, $[B_1,B_2,B_3,B_4]=[ 1, 0, 1, 0]$ then Eq. (29) changes into,

37 $$\begin{aligned} \Lambda (\rho )=\frac{\exp (\rho )}{\exp (\rho )+1}. \end{aligned}$$

Substituting Eq. (30) with the help of Eq. (37) into the Eq. (27) the set of equations is obtained. Using Mathematica to solve this set of equations will yield the following unknown constants such as:

- *Set 1* The unknown constants are $a_0=2 l \nu ,a_1=-2 l \nu , b_1=0,c=l^2 \nu ,$ the stochastic exponential function solution is obtained for Eq. (1) such as,
  38 $$\begin{aligned} \Upsilon _{5}(x,t)=\left( 2 l \nu -\frac{2 l \nu e^{l x-l^2 \nu t}}{e^{l x-l^2 \nu t}+1}\right) e^{\sigma \beta (t)-\frac{\sigma ^2}{2}t}. \end{aligned}$$

### Family of solution 4

If $[A_1, A_2, A_3,A_4]=[ 1-i, 1+i, 1, 1]$, $[B_1,B_2,B_3,B_4]= [i, -i, i, -i]$ then Eq. (29) changes into,

39 $$\begin{aligned} \Lambda (\rho )=\frac{\sin (\rho )+\cos (\rho )}{\cos (\rho )}. \end{aligned}$$

Substituting Eq. (30) with the help of Eq. (39) into the Eq. (27) the set of equations is obtained. Using Mathematica to solve this set of equations will yield the following unknown constants such as:

- *Set 1* The unknown constants are $a_0=(-2-2 i) l \nu ,a_1=2 l \nu , b_1=0,c=-2 i l^2 \nu$, the stochastic SWS is obtained for Eq. (1) such as,
  40 $$\begin{aligned} \Upsilon _{6}(x,t)=\left( 2 l \nu \sec \left( l x+2 i l^2 \nu t\right) \left( \sin \left( l x+2 i l^2 \nu t\right) +\cos \left( l x+2 i l^2 \nu t\right) \right) +(-2-2 i) l \nu \right) e^{\sigma \beta (t)-\frac{\sigma ^2}{2}t}. \end{aligned}$$
- *Set 2* The unknown constants are $a_0=(-2+2 i) l \nu ,a_1=2 l \nu , b_1=0,c=2 i l^2 \nu ,$ the stochastic SWS is obtained for Eq. (1) such as,
  41 $$\begin{aligned} \Upsilon _{7}(x,t)=\left( 2 l \nu \sec \left( l x-2 i l^2 \nu t\right) \left( \sin \left( l x-2 i l^2 \nu t\right) +\cos \left( l x-2 i l^2 \nu t\right) \right) +(-2+2 i) l \nu \right) e^{\sigma \beta (t)-\frac{\sigma ^2}{2}t}. \end{aligned}$$

### Family of solution 5

If $[A_1, A_2, A_3,A_4]=[- 3, - 1, 1, 1]$, $[B_1,B_2,B_3,B_4]= [1, - 1, 1, - 1]$ then Eq. (29) changes into,

42 $$\begin{aligned} \Lambda (\rho )=\frac{-\sinh (\rho )-2 \cosh (\rho )}{\cosh (\rho )}. \end{aligned}$$

Substituting Eq. (30) with the help of Eq. (42) into the Eq. (27) the set of equations is obtained. Using Mathematica to solve this set of equations will yield the following unknown constants such as:

- *Set 1* The unknown constants are $a_0=-2 l \nu ,a_1=0 , b_1=-6 l \nu ,c=2 l^2 \nu ,$ the stochastic soliton solution is obtained for Eq. (1) such as,
  43 $$\begin{aligned} \Upsilon _{8}(x,t)=\left( -\frac{6 l \nu \cosh \left( l x-2 l^2 \nu t\right) }{-\sinh \left( l x-2 l^2 \nu t\right) -2 \cosh \left( l x-2 l^2 \nu t\right) }-2 l \nu \right) e^{\sigma \beta (t)-\frac{\sigma ^2}{2}t}. \end{aligned}$$
- *Set 2* The unknown constants are $a_0=2 l \nu ,a_1=2 l \nu ,b_1=0 ,c=-2 l^2 \nu ,$ the stochastic soliton solution is obtained for Eq. (1) such as,
  44 $$\begin{aligned} \Upsilon _{9}(x,t)=\left( 2 l \nu \text {sech}\left( 2 l^2 \nu t+l x\right) \left( -\sinh \left( 2 l^2 \nu t+l x\right) -2 \cosh \left( 2 l^2 \nu t+l x\right) \right) +2 l \nu \right) e^{\sigma \beta (t)-\frac{\sigma ^2}{2}t}. \end{aligned}$$

### Family of solution 6

If $[A_1, A_2, A_3,A_4]=[-1, 0, 1, 1]$, $[B_1,B_2,B_3,B_4]= [0, 1, 0, 1]$ then Eq. (29) changes into,

45 $$\begin{aligned} \Lambda (\rho )=-\frac{1}{\exp (\rho )+1}. \end{aligned}$$

Substituting Eq. (30) with the help of Eq. (45) into the Eq. (27) the set of equations is obtained. Using Mathematica to solve this set of equations will yield the following unknown constants such as:

- *Set 1* The unknown constants are $a_0=\frac{\text {c2}}{l} ,a_1=2 l \nu , b_1=0 ,c=l^2 \nu -\text {c2},$ the stochastic exponential function solution is obtained for Eq. (1) such as,
  46 $$\begin{aligned} \Upsilon _{10}(x,t)=\left( \frac{\text {c2}}{l}-\frac{2 l \nu }{e^{l x-t \left( l^2 \nu -\text {c2}\right) }+1}\right) e^{\sigma \beta (t)-\frac{\sigma ^2}{2}t}. \end{aligned}$$

### Family of solution 7

If $[A_1, A_2, A_3,A_4]=[ 1, 0, 1, 1]$, $[B_1,B_2,B_3,B_4]= [1, 0, 1, 0]$ then Eq. (29) changes into,

47 $$\begin{aligned} \Lambda (\rho )=-\frac{\sinh (\rho )}{\cosh (\rho )}. \end{aligned}$$

Substituting Eq. (30) with the help of Eq. (47) into the Eq. (27) the set of equations is obtained. Using Mathematica to solve this set of equations will yield the following unknown constants such as:

- *Set 1* The unknown constants are $a_0=2 l \nu ,a_1=2 l \nu ,b_1=0 ,c=2 l^2 \nu ,$ the dark stochastic soliton solution is obtained for Eq. (1) such as,
  48 $$\begin{aligned} \Upsilon _{11}(x,t)=\left( 2 l \nu -2 l \nu \tanh \left( l x-2 l^2 \nu t\right) \right) e^{\sigma \beta (t)-\frac{\sigma ^2}{2}t}. \end{aligned}$$
- *Set 2* The unknown constants are $a_0=-4 l \nu ,a_1=2 l \nu ,b_1=2 l \nu ,c=- 4 l^2 \nu ,$ the stochastic soliton solution is obtained for Eq. (1) such as,
  49 $$\begin{aligned} \Upsilon _{12}(x,t)=\left( -2 l \nu \tanh \left( 4 l^2 \nu t+l x\right) -2 l \nu \coth \left( 4 l^2 \nu t+l x\right) -4 l \nu \right) e^{\sigma \beta (t)-\frac{\sigma ^2}{2}t}. \end{aligned}$$

### Family of solution 8

If $[A_1, A_2, A_3,A_4]=[2-i, 2+i, 1, 1]$, $[B_1,B_2,B_3,B_4]= [ i, -i, i, -i]$ then Eq. (29) changes into,

50 $$\begin{aligned} \Lambda (\rho )=\frac{\sin (\rho )+2 \cos (\rho )}{\cos (\rho )}. \end{aligned}$$

Substituting Eq. (30) with the help of Eq. (50) into the Eq. (27) the set of equations is obtained. Using Mathematica to solve this set of equations will yield the following unknown constants such as:

- *Set 1* The unknown constants are $a_0=(4+2 i) l \nu , a_1=0 ,b_1=-10 l \nu ,c=2 i l^2 \nu ,$ the stochastic SWS of Eq. (1) is obtained as,
  51 $$\begin{aligned} \Upsilon _{13}(x,t)=\left( (4+2 i) l \nu -\frac{10 l \nu \cos \left( l x-2 i l^2 \nu t\right) }{\sin \left( l x-2 i l^2 \nu t\right) +2 \cos \left( l x-2 i l^2 \nu t\right) }\right) e^{\sigma \beta (t)-\frac{\sigma ^2}{2}t}. \end{aligned}$$
- *Set 2* The unknown constants are $a_0=(-4-2 i) l \nu , a_1=2 l \nu ,b_1=0 ,c=-2 i l^2 \nu ,$ the stochastic SWS of Eq. (1) is obtained as,
  52 $$\begin{aligned} \Upsilon _{14}(x,t)=\left( 2 l \nu \sec \left( l x+2 i l^2 \nu t\right) \left( \sin \left( l x+2 i l^2 \nu t\right) +2 \cos \left( l x+2 i l^2 \nu t\right) \right) +(-4-2 i) l \nu \right) e^{\sigma \beta (t)-\frac{\sigma ^2}{2}t}. \end{aligned}$$

### Family of solution 9

If $[A_1, A_2, A_3,A_4]=[ 1 , 2 , 1 , 1]$, $[B_1,B_2,B_3,B_4]=[ 1, 0, 1, 0]$ then Eq. (29) changes into,

53 $$\begin{aligned} \Lambda (\rho )=\frac{\exp (\rho )+2}{\exp (\rho )+1}. \end{aligned}$$

Substituting Eq. (30) with the help of Eq. (53) into the Eq. (27) the set of equations is obtained. Using Mathematica to solve this set of equations will yield the following unknown constants such as:

- *Set 1* The unknown constants are $a_0=4 l \nu ,a_1=0 ,b_1=-4 l \nu ,c=l^2 \nu ,$ the stochastic exponential function solution is obtained for Eq. (1) such as,
  54 $$\begin{aligned} \Upsilon _{15}(x,t)=\left( 4 l \nu -\frac{4 l \nu \left( e^{l x-l^2 \nu t}+1\right) }{e^{l x-l^2 \nu t}+2}\right) e^{\sigma \beta (t)-\frac{\sigma ^2}{2}t}. \end{aligned}$$
- *Set 2* The unknown constants are $a_0=-4 l \nu ,a_1=2 l \nu ,b_1=0 ,c=-l^2 \nu ,$ the stochastic exponential function solution is obtained for Eq. (1) such as,
  55 $$\begin{aligned} \Upsilon _{16}(x,t)=\left( \frac{2 l \nu \left( e^{l^2 \nu t+l x}+2\right) }{e^{l^2 \nu t+l x}+1}-4 l \nu \right) e^{\sigma \beta (t)-\frac{\sigma ^2}{2}t}. \end{aligned}$$

### Family of solution 10

If $[A_1, A_2, A_3,A_4]=[2, 1, 1, 1]$, $[B_1,B_2,B_3,B_4]=[1 , 0 , 1 , 0]$ then Eq. (29) changes into,

56 $$\begin{aligned} \Lambda (\rho )=\frac{\exp (\rho )}{\exp (\rho )+1}. \end{aligned}$$

Substituting Eq. (30) with the help of Eq. (56) into the Eq. (27) the set of equations is obtained. Using Mathematica to solve this set of equations will yield the following unknown constants such as:

- *Set 1* The unknown constants are $a_0=-2 l \nu ,a_1=0 ,b_1=4 l \nu ,c=l^2 \nu ,$ the stochastic exponential function solution is obtained for Eq. (1) such as,
  57 $$\begin{aligned} \Upsilon _{17}(x,t)=\left( \frac{4 l \nu \left( e^{l x-l^2 \nu t}+1\right) }{2 e^{l x-l^2 \nu t}+1}-2 l \nu \right) e^{\sigma \beta (t)-\frac{\sigma ^2}{2}t}. \end{aligned}$$
- *Set 2* The unknown constants are $a_0=2 l \nu ,a_1=-2 l \nu ,b_1=0 ,c=-l^2 \nu ,$ the stochastic exponential function solution is obtained for Eq. (1) such as,
  58 $$\begin{aligned} \Upsilon _{18}(x,t)=\left( 2 l \nu -\frac{2 l \nu \left( e^{l^2 \nu t+l x}+2\right) }{e^{l^2 \nu t+l x}+1}\right) e^{\sigma \beta (t)-\frac{\sigma ^2}{2}t}. \end{aligned}$$

### Family of solution 11

If $$ [A_1, A_2, A_3,A_4]=[1, 1, 1, 1]$$, $$[B_1,B_2,B_3,B_4]=[ 0, 0, 1, -1]$$ then Eq. (29) changes into,

59 $$\begin{aligned} \Lambda (\rho )=\frac{2}{\exp (-\rho )+\exp (\rho )}. \end{aligned}$$

Substituting Eq. (30) with the help of Eq. (59) into the Eq. (27) the set of equations is obtained. Using Mathematica to solve this set of equations will yield the following unknown constants such as:

- *Set 1* The unknown constants are $a_0=-\frac{1}{7} (26 l \nu ), a_1=\frac{2}{7} \sqrt{13} l \nu ,b_1=\frac{4}{7} \sqrt{26} l \nu ,c=\frac{1}{7} \left( -4 \sqrt{2} l^2 \nu -21 l^2 \nu \right) ,$ the stochastic exponential function solution is obtained for Eq. (1) such as,
  60 $$\begin{aligned} \Upsilon _{19}(x,t)=\left( -\frac{26 l \nu }{7}+\frac{2}{7} \sqrt{26} l \nu \left( e^{l x-\frac{1}{7} t \left( -4 \sqrt{2} l^2 \nu -21 l^2 \nu \right) }+e^{\frac{1}{7} t \left( -4 \sqrt{2} l^2 \nu -21 l^2 \nu \right) -l x}\right) \right. \nonumber \\ \left. +\frac{4 \sqrt{13} l \nu }{7 \left( e^{l x-\frac{1}{7} t \left( -4 \sqrt{2} l^2 \nu -21 l^2 \nu \right) }+e^{\frac{1}{7} t \left( -4 \sqrt{2} l^2 \nu -21 l^2 \nu \right) -l x}\right) }\right) e^{\sigma \beta (t)-\frac{\sigma ^2}{2}t}. \end{aligned}$$

## Results and discussion

This section presents the results that are extracted successfully by the numerical and analytical techniques and their graphical comparison as well. The numerical solutions are gained from the proposed SFD scheme (12) and compared with different stochastic ESW solutions that are successfully constructed by using the GERF method. These solutions are verified with the help of Mathematica 11.1. The different forms of solutions are constructed in the form of hyperbolic, trigonometric, and exponential function solutions.

### Exact solitary wave solutions

**Figure 2:**
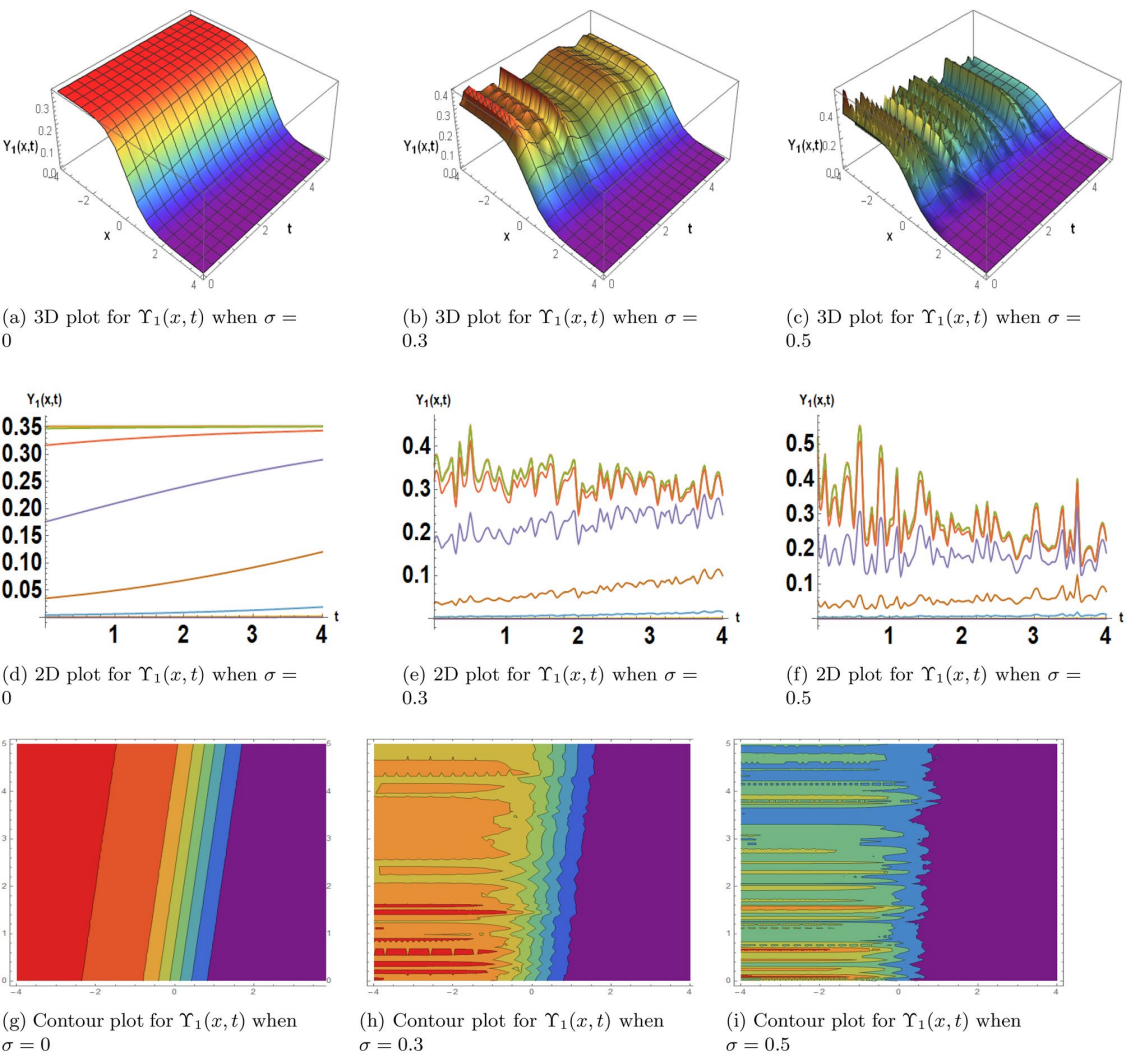
The subfigures (**a–c**) shows the 3D, subfigures (**d–f**) shows the 2D, and subfigures (**g–i**) shows the contours for the solution $\Upsilon _1(x,t)$ under the different noise strengths.

**Figure 3:**
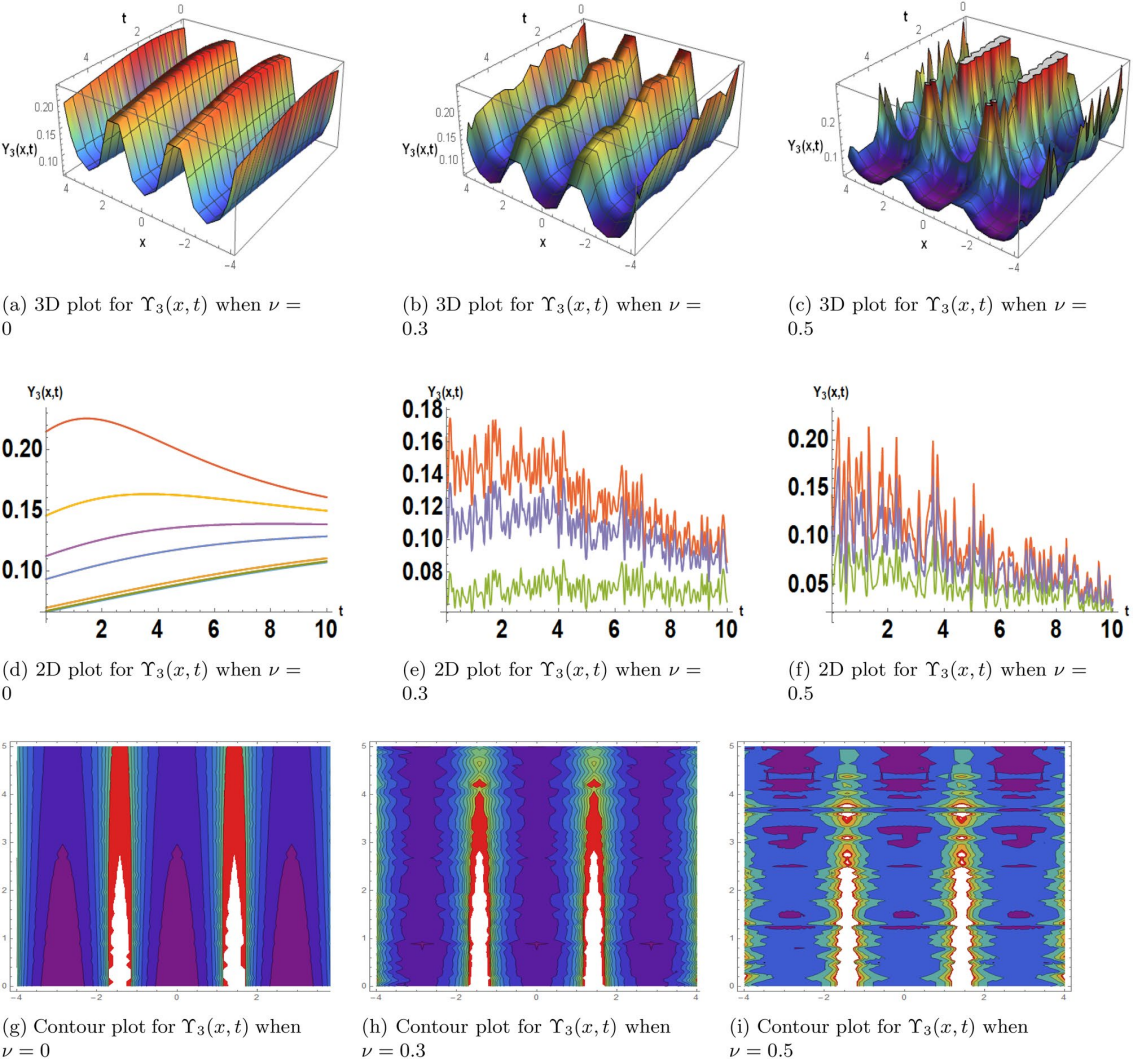
The subfigures (**a–c**) shows the 3D, subfigures (**d–f**) shows the 2D, and subfigures (**g–i**) shows the contours for the solution $\Upsilon _3(x,t)$ under the different noise strengths.

**Figure 4:**
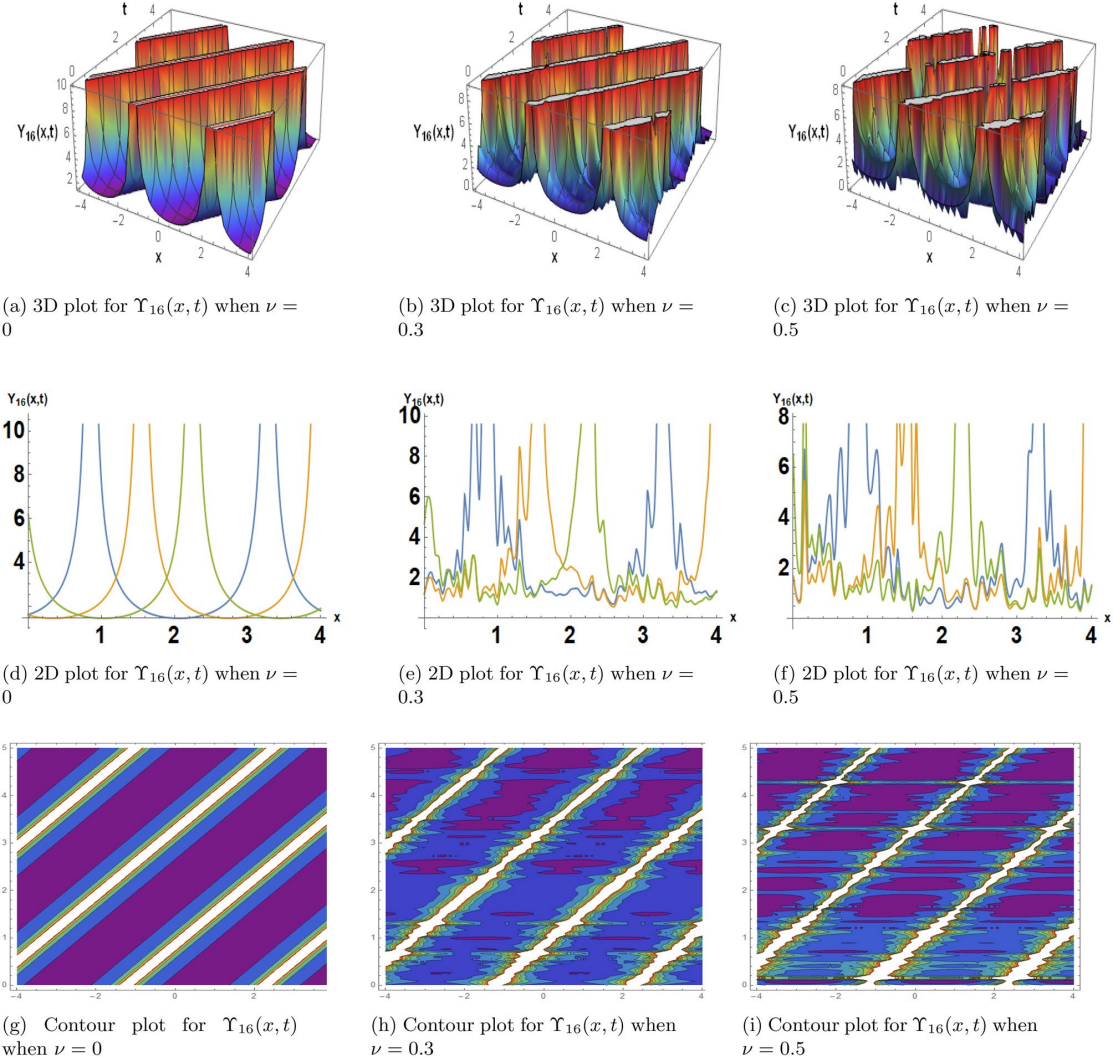
The subfigures (**a–c**) shows the 3D, subfigures (**d–f**) shows the 2D, and subfigures (**g–i**) shows the contours for the solution $\Upsilon _{16}(x,t)$ under the different noise strengths.

This subsection, presents the exact solitary wave solutions under the different effects of noise. Numerous real-world applications, particularly in physics, engineering, and other domains, include solitary waves and solitons in the framework of the stochastic Burgers model under noise. A partial differential equation representing the development of a one-dimensional random field, the stochastic Burgers equation is a deterministic Burgers equation extended by stochastic noise. Solitons are useful for modeling and comprehending turbulence and fluid dynamics. When random fluctuations are present, the stochastic Burgers equation with soliton solutions aids in forecasting the behavior of waves and disturbances in situations like river flows or ocean currents. Soliton solutions may reflect coherent structures or traffic jams in the context of studying traffic flow using the stochastic Burgers model. A more accurate depiction of uncertainty and unpredictability in traffic patterns is made possible by the models incorporation of noise. Solitons are important for studying the interactions and propagation of waves in plasma physics. One tool for analyzing the impact of random perturbations on soliton stability in plasmas is the stochastic Burgers equation under noise. The Fig. 2 is drawn for the solutions $\Upsilon _1(x,t)$ that with provide the dark soliton solution. The Figs. 3 and 4 are drawn for the solutions $\Upsilon _3(x,t)$ and $\Upsilon _{16}(x,t)$ respectively that are clearly provided us the solitary wave solutions. The different 3D, 2D and its corresponding contour plots are drawn. These plot are drawn for the different effects of noise by choosing different values of $\sigma .$ When we choose $\sigma$ zero these solitary wave solutions or soliton solutions are the classical but when we increase the value of $\sigma$ these plots are shows the randomness in there behavior.

### Comparison of results

**Figure 5:**
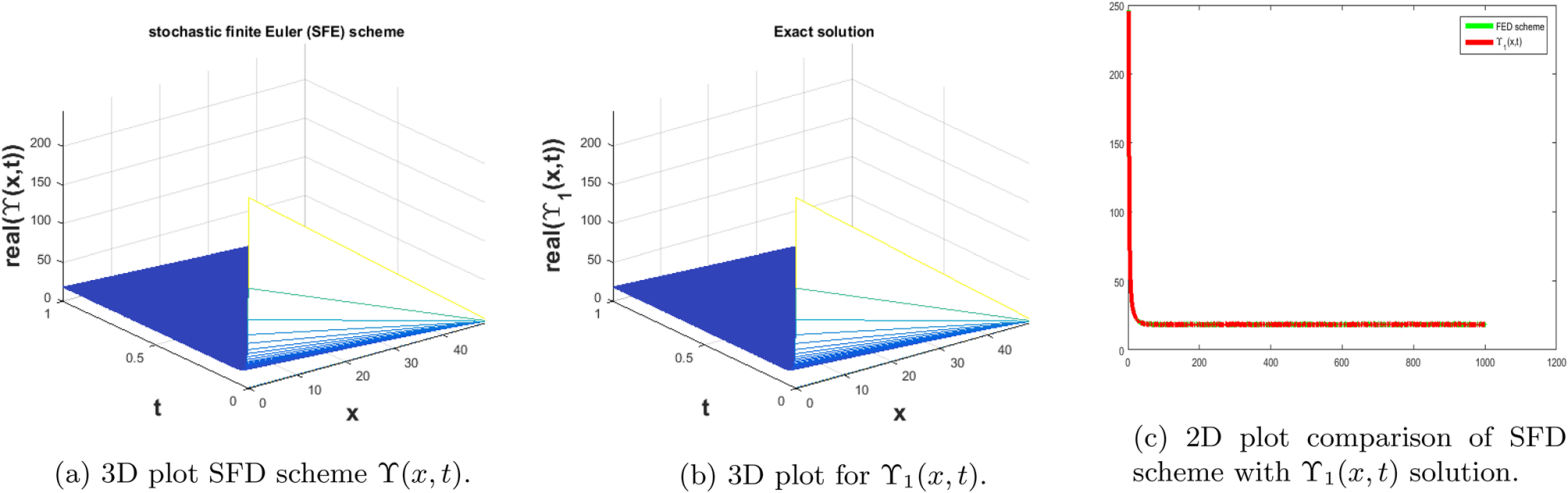
The subfigure (**a**) shows the 3D behavior of SFD scheme, subfigure (**b**) shows the stochastic exact solitary wave solution while subfigure (**c**) shows the 2D comparison of SFD scheme with exact solitary wave solution.

**Figure 6:**
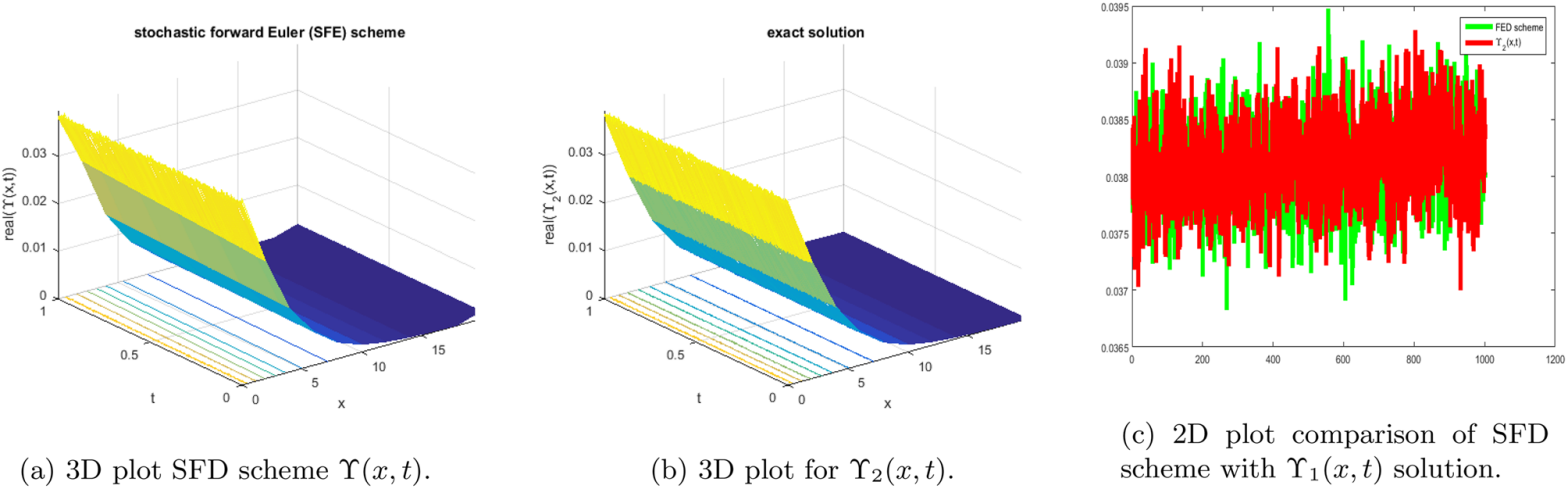
The subfigure (**a**) shows the 3D behavior of SFD scheme, subfigure (**b**) shows the stochastic exact solitary wave solution while subfigure (**c**) shows the 2D comparison of SFD scheme with exact solitary wave solution.

**Figure 7:**
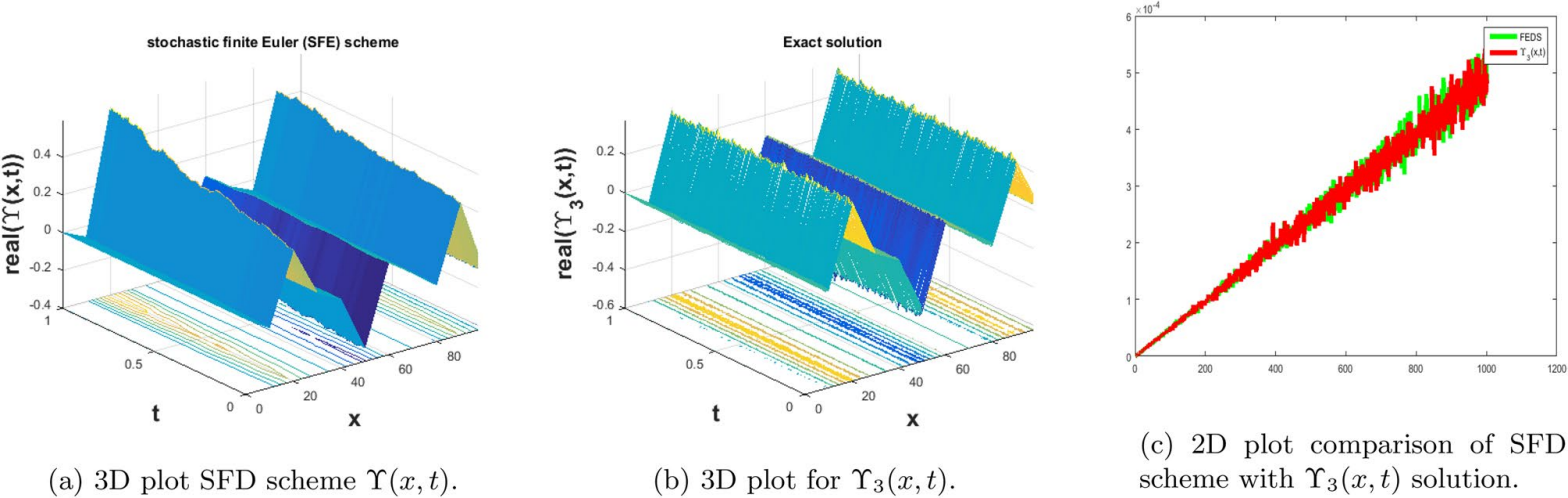
The subfigure (**a**) shows the 3D behavior of SFD scheme, subfigure (**b**) shows the stochastic exact solitary wave solution while subfigure (**c**) shows the 2D comparison of SFD scheme with exact solitary wave solution.

**Figure 8:**
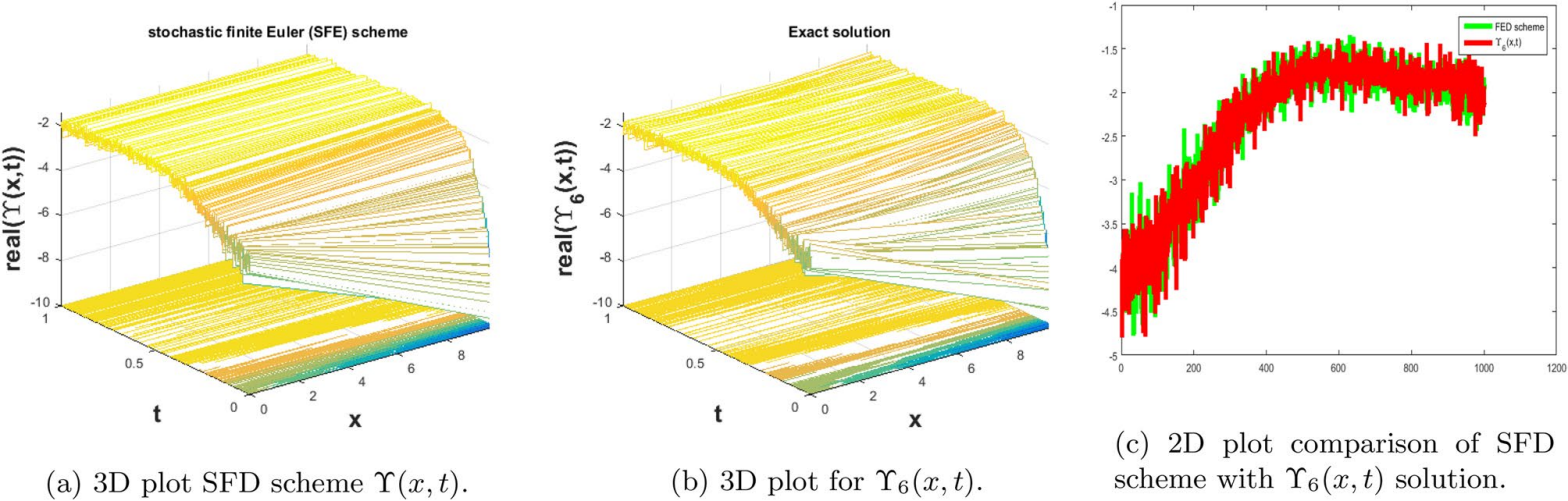
The subfigure (**a**) shows the 3D behavior of SFD scheme, subfigure (**b**) shows the stochastic exact solitary wave solution while subfigure (**c**) shows the 2D comparison of SFD scheme with exact solitary wave solution.

**Figure 9:**
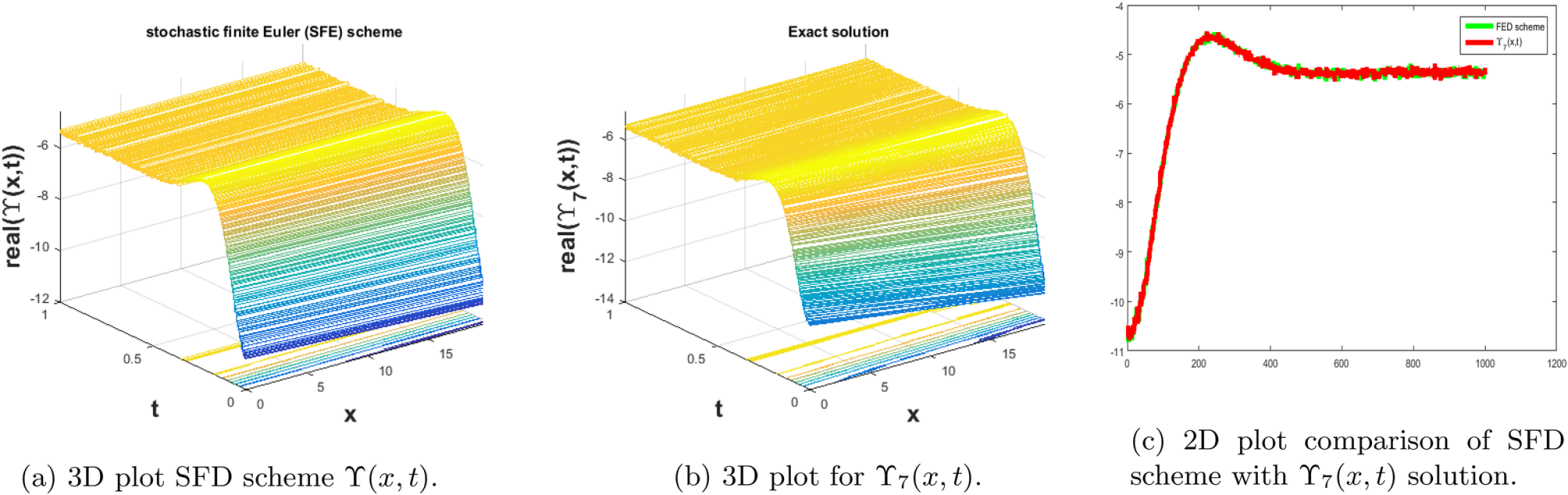
The subfigure (**a**) shows the 3D behavior of SFD scheme, subfigure (**b**) shows the stochastic exact solitary wave solution while subfigure (**c**) shows the 2D comparison of SFD scheme with exact solitary wave solution.

**Figure 10:**
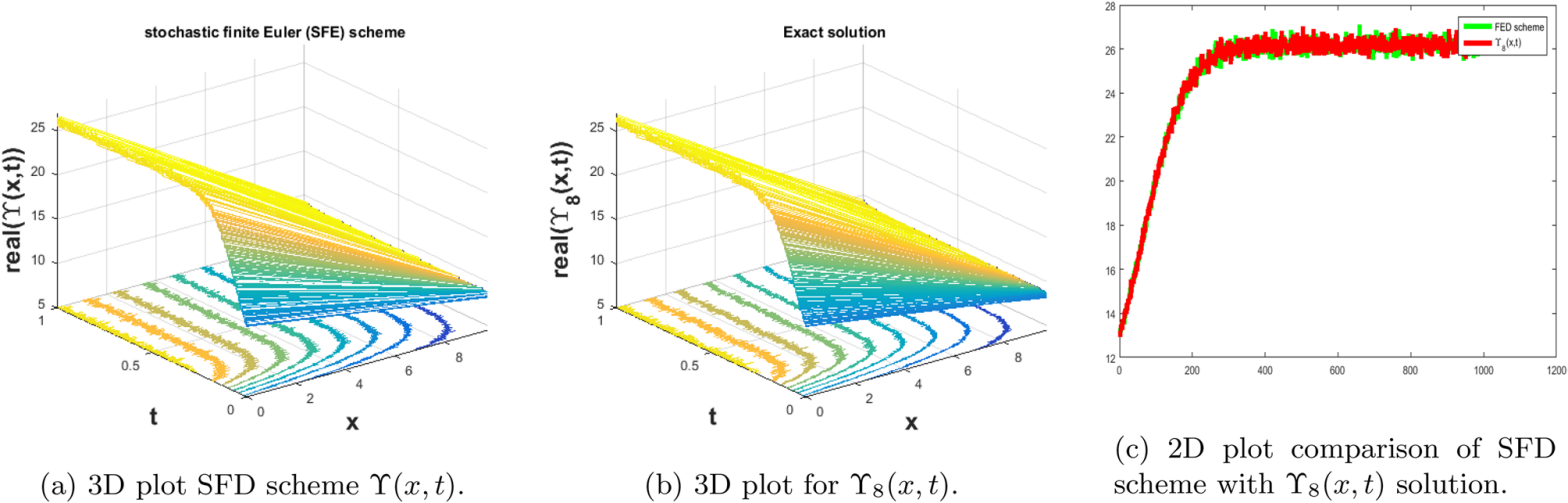
The subfigure (**a**) shows the 3D behavior of SFD scheme, subfigure (**b**) shows the stochastic exact solitary wave solution while subfigure (**c**) shows the 2D comparison of SFD scheme with exact solitary wave solution.

In this subsection, we give a comparison of some newly constructed stochastic exact solutions with the SFD scheme. Where $\sigma$ is the control parameter of Brownian motion, *l* and $\nu$ are unknown constants while *h* is the time step size while *k* is the space step size. The space step size $k=10^{-3}$ is fixed for all plots while other parameters are varies for different plots. The newly constructed singular stochastic soliton $\Upsilon _1(x, t)$ in Eq. (32) is compare with proposed SFD scheme by selecting the $\sigma =0.05, l=4.9, \nu =0.1, h=50$. By selecting these parameters both results are gives us the same behavior graphically in t he 2D and line plots that are dispatched in Fig. 5. For the Fig. 6 we consider the $\Upsilon _2(x, t)$ in Eq. (33) and select the parameters such as $\sigma =0.02, l=0.19, \nu =0.1, h=2$, while Fig. 7 is drawn for the solution $\Upsilon _3(x, t)$ in Eq. (35) and choose the parameters as follows $\sigma =0.05, l=0.09, \nu =0.41, h=10$. The Figs. 8, 9 and 10 are drawn for the exact solutions $\Upsilon _6(x, t)$, $\Upsilon _7(x, t)$, $\Upsilon _8(x, t)$ and their corresponding parameters are $\sigma =0.09, l=1.9999, \nu =0.5, h=10$, $\sigma =0.01, l=1.9, \nu =1.41, h=20,$ and $\sigma =0.01, l=0.99, \nu =4.41, h=10$, respectively. At the end considering the solution $\Upsilon _{11}(x, t)$, $\Upsilon _{13}(x, t)$ and draw the Figs. 12 and 13 and which are provided us the same behavior for the numerical and exact solutions that are clearly shown in 3D and line graphs as well. This study is very helpful and fruitful for the dynamical systems under the influence of randomness.

#### Problem 1

For the comparison of numerical result by proposed SFD scheme (12) with stochastic exact solitary wave solutions Eq. (32). The Fig. 5 is shown a similar behavior for the computational results with exact solitary wave solutions $\Upsilon _1(x,t)$ under the influence of randomness which is clearly shown physically. To, compare numerical results we construct the ICs and BCs such as

61 $$\begin{aligned} \Upsilon (x, 0)=0.0377345\, - 0.0377345 \coth (0.19 x), \end{aligned}$$

the BCs are as follows,

62 $$\begin{aligned} \Upsilon (0, t)= & {} e^{- 0.0002 t} (0.0377345 \coth (0.00722 t)+0.0377345), \end{aligned}$$

63 $$\begin{aligned} \Upsilon (10, t)= & {} e^{- 0.0002 t} (0.0377345\, - 0.0377345 \coth (1.9\, - 0.00722 t)). \end{aligned}$$

#### Problem 2

For the comparison of numerical result by proposed SFD scheme (12) with stochastic exact solitary wave solutions Eq. (33). The Fig. 6 is shown a similar behavior for the computational results with exact solitary wave solutions $\Upsilon _2(x,t)$ under the influence of randomness which is clearly shown physically. To, compare numerical results we construct the ICs and BCs such as

64 $$\begin{aligned} \Upsilon (x, 0)=0.0385277\, - 0.0385277 \tanh (0.19 x), \end{aligned}$$

the BCs are as follows,

65 $$\begin{aligned} \Upsilon (0, t)= & {} e^{- 0.0002 t} (0.0385277 \tanh (0.00722 t)+0.0385277), \end{aligned}$$

66 $$\begin{aligned} \Upsilon (10, t)= & {} e^{- 0.0002 t} (0.0385277\, - 0.0385277 \tanh (1.9\, - 0.00722 t)). \end{aligned}$$

#### Problem 3

For the comparison of numerical result by proposed SFD scheme (12) with stochastic exact solitary wave solutions Eq. (35). The Fig. 7 is shown a similar behavior for the computational results with exact solitary wave solutions $\Upsilon _3(x,t)$ under the influence of randomness which is clearly shown physically. To, compare numerical results we construct the ICs and BCs such as

67 $$\begin{aligned} \Upsilon (x, 0)= - 0.0793009 \tan (0.09 x)+(0.0793009 i), \end{aligned}$$

the BCs are as follows,

68 $$\begin{aligned} \Upsilon (0, t)= & {} e^{- 0.00125 t} ((0.\, + 0.0793009 i) \tanh (0.006642 t)+(0.\, +0.0793009 i)), \end{aligned}$$

69 $$\begin{aligned} \Upsilon (10, t)= & {} e^{- 0.00125 t} ((0.\, + 0.0793009 i)-0.0793009 \tan (0.9\, - (0.\, + 0.006642 i) t)). \end{aligned}$$

#### Problem 4

For the comparison of numerical result by proposed SFD scheme (12) with stochastic exact solitary wave solutions Eq. (40). The Fig. 8 is shown a similar behavior for the computational results with exact solitary wave solutions $\Upsilon _6(x,t)$ under the influence of randomness which is clearly shown physically. To, compare numerical results we construct the ICs and BCs such as

70 $$\begin{aligned} \Upsilon (x, 0)=1.90456 \tan (1.9999 x)+(0.\, -1.90456 i), \end{aligned}$$

the BCs are as follows,

71 $$\begin{aligned} \Upsilon (0, t)= & {} e^{-0.00405 t} ((0.\, +1.90456 i) \tanh (3.9996 t)+(0.\, -1.90456 i)), \end{aligned}$$

72 $$\begin{aligned} \Upsilon (10, t)= & {} e^{-0.00405 t} (1.90456 \tan (19.999+(0.\, +3.9996 i) t)+(0.\, -1.90456 i)). \end{aligned}$$

#### Problem 5

For the comparison of numerical result by proposed SFD scheme (12) with stochastic exact solitary wave solutions Eq. (41). The Fig. 9 is shown a similar behavior for the computational results with exact solitary wave solutions $\Upsilon _7(x,t)$ under the influence of randomness which is clearly shown physically. To, compare numerical results we construct the ICs and BCs such as

73 $$\begin{aligned} \Upsilon (x, 0)=5.32506 \tan (1.9 x)+(0.\, +5.32506 i), \end{aligned}$$

the BCs are as follows,

74 $$\begin{aligned} \Upsilon (0, t)= & {} e^{- 0.00005 t} ((5.32506 i) - (5.32506 i) \tanh (10.1802 t)), \end{aligned}$$

75 $$\begin{aligned} \Upsilon (10, t)= & {} e^{- 0.00005 t} (5.32506 \tan (19.\, - (10.1802 i) t) + (5.32506 i)). \end{aligned}$$

#### Problem 6

For the comparison of numerical result by proposed SFD scheme (12) with stochastic exact solitary wave solutions Eq. (43). The Fig. 10 is shown a similar behavior for the computational results with exact solitary wave solutions $\Upsilon _8(x,t)$ under the influence of randomness which is clearly shown physically. To, compare numerical results we construct the ICs and BCs such as

76 $$\begin{aligned} \Upsilon (x, 0)=\big (0.0578492\, +0.173548 e^{1.98 x}\big )^{-1}, \end{aligned}$$

the BCs are as follows,

77 $$\begin{aligned} \Upsilon (0, t)= & {} \big (0.173548 e^{-17.2889 t}+0.0578492 e^{0.00005 t}\big )^{-1}, \end{aligned}$$

78 $$\begin{aligned} \Upsilon (10, t)= & {} \big (6.89365\times 10^7 e^{-17.2889 t}+0.0578492 e^{0.00005 t}\big )^{-1}. \end{aligned}$$

#### Problem 7

**Figure 11:**
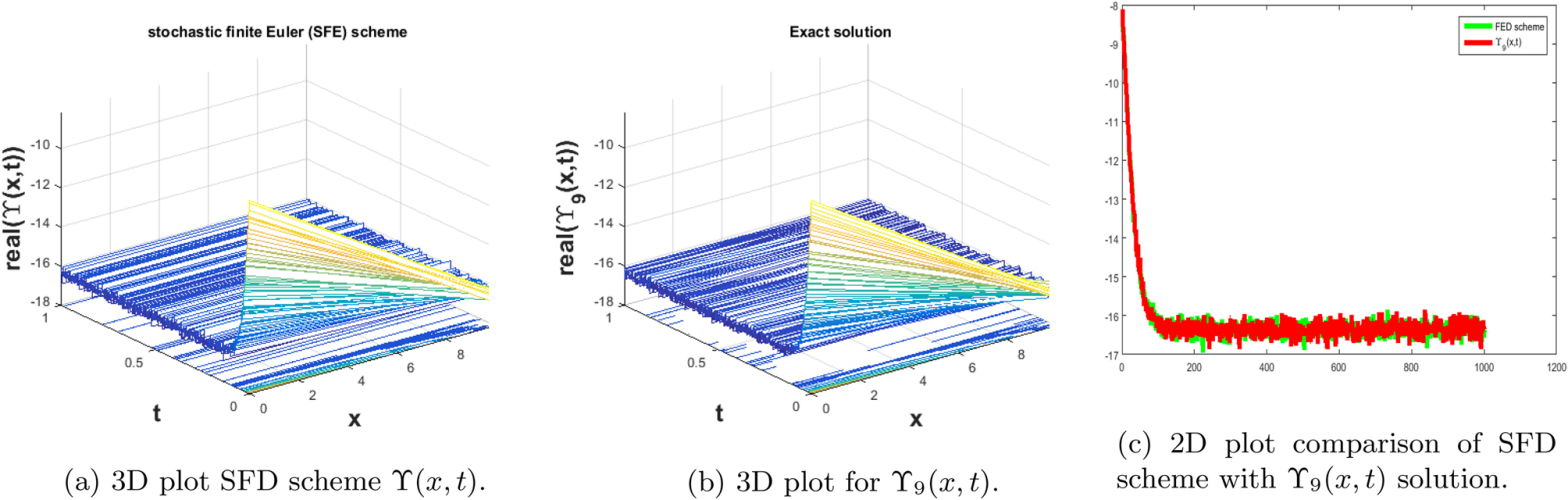
The subfigure (**a**) shows the 3D behavior of SFD scheme, subfigure (**b**) shows the stochastic exact solitary wave solution while subfigure (**c**) shows the 2D comparison of SFD scheme with exact solitary wave solution.

For the comparison of numerical result by proposed SFD scheme (12) with stochastic exact solitary wave solutions Eq. (44). The Fig. 11 is shown a similar behavior for the computational results with exact solitary wave solutions $\Upsilon _9(x,t)$ under the influence of randomness which is clearly shown physically. To, compare numerical results we construct the ICs and BCs such as

79 $$\begin{aligned} \Upsilon (x, 0) = - 8.22152 \tanh (2.9 x) - 8.22152, \end{aligned}$$

the BCs are as follows,

80 $$\begin{aligned} \Upsilon (0, t)= & {} e^{- 0.00005 t} ({-} 8.22152 \tanh (23.7162 t) - 8.22152), \end{aligned}$$

81 $$\begin{aligned} \Upsilon (10, t)= & {} e^{- 0.00005 t} ({-} 8.22152 \tanh (23.7162 t+29.) - 8.22152). \end{aligned}$$

#### Problem 8

**Figure 12:**
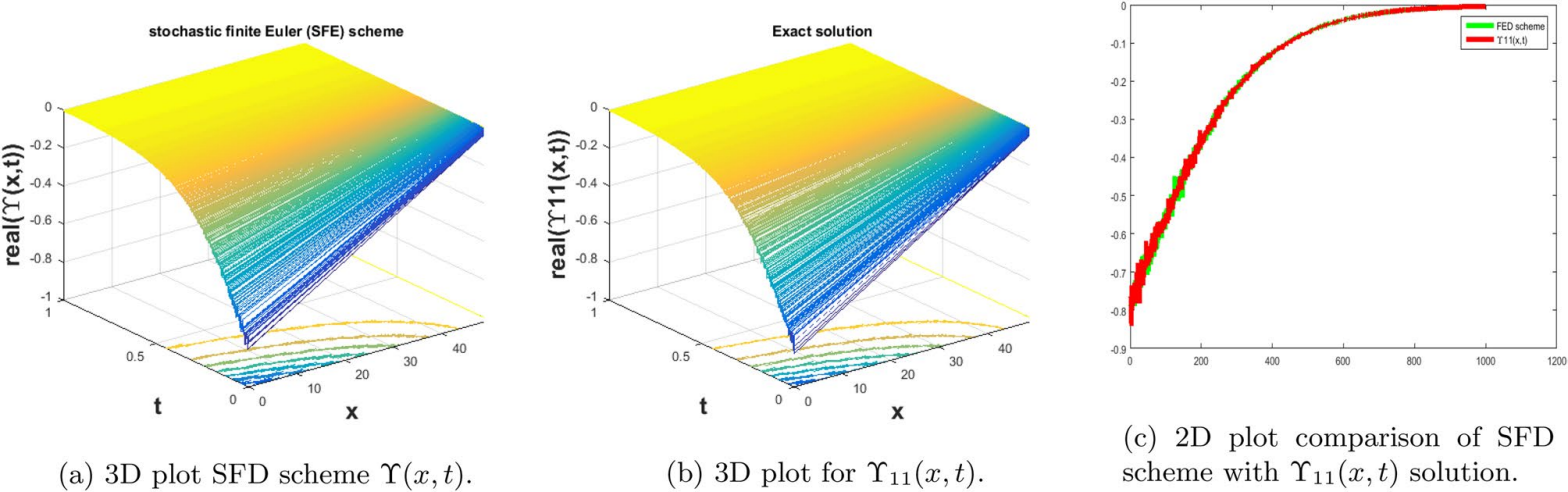
The subfigure (**a**) shows the 3D behavior of SFD scheme, subfigure (**b**) shows the stochastic exact solitary wave solution while subfigure (**c**) shows the 2D comparison of SFD scheme with exact solitary wave solution.

For the comparison of numerical result by proposed SFD scheme (12) with stochastic exact solitary wave solutions Eq. (48). Figure 12 is shown a similar behavior for the computational results with exact solitary wave solutions $\Upsilon _{11}(x,t)$ under the influence of randomness which is clearly shown physically. To, compare numerical results we construct the ICs and BCs such as

82 $$\begin{aligned} \Upsilon (x, 0)=0.853644 \tanh (3.9 x)-0.853644, \end{aligned}$$

the BCs are as follows,

83 $$\begin{aligned} \Upsilon (0, t)= & {} e^{-0.00045 t} (0.853644 \tanh (3.042 t)-0.853644), \end{aligned}$$

84 $$\begin{aligned} \Upsilon (10, t)= & {} e^{-0.00045 t} (0.853644 \tanh (3.042 t+39.)-0.853644). \end{aligned}$$

#### Problem 9

**Figure 13:**
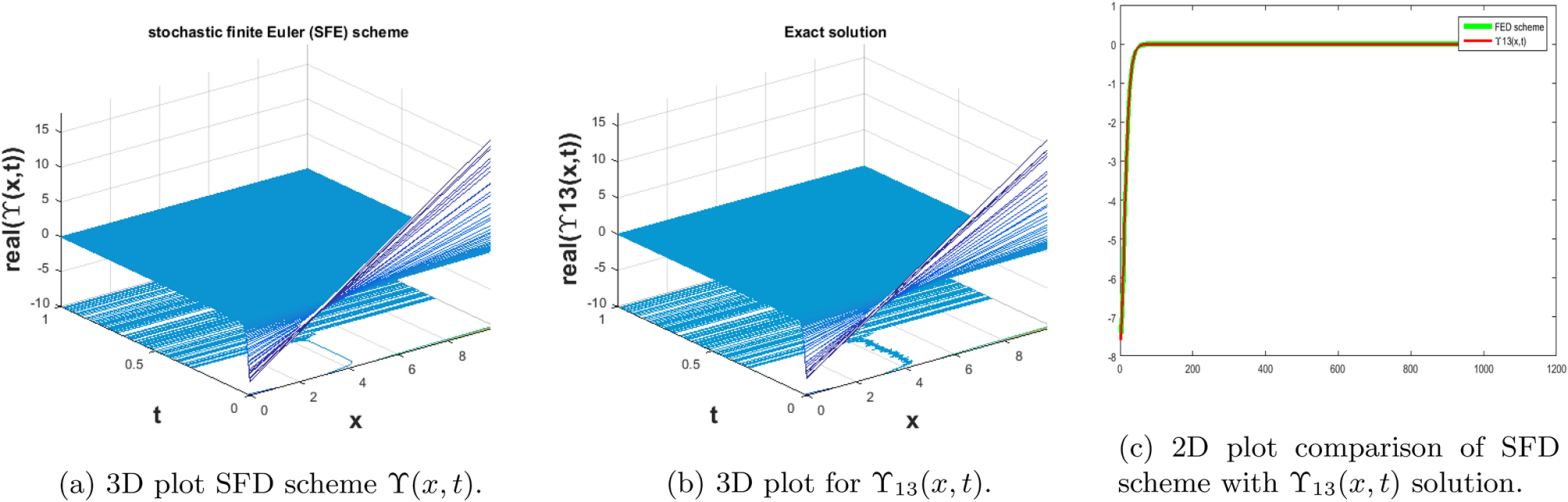
The subfigure (**a**) shows the 3D behavior of SFD scheme, subfigure (**b**) shows the stochastic exact solitary wave solution while subfigure (**c**) shows the 2D comparison of SFD scheme with exact solitary wave solution.

For the comparison of numerical result by proposed SFD scheme (12) with stochastic exact solitary wave solutions Eq. (51). Figure 13 is shown a similar behavior for the computational results with exact solitary wave solutions $\Upsilon _{13}(x,t)$ under the influence of randomness which is clearly shown physically. To, compare numerical results we construct the ICs and BCs such as

85 $$\begin{aligned} \Upsilon (x, 0)=(-0.0129729 \tan (3.9 x)-0.0259459)^{-1}+(30.8334\, +15.4167 i), \end{aligned}$$

the BCs are as follows,

86 $$\begin{aligned} \Upsilon (0, t)= & {} \frac{e^{-0.00045 t} \bigg ((1.4210854715202005^{-15}+7.105427357601002^{-16} i) e^{116.204 t}+(-24.6667+18.5 i)\bigg )}{1. e^{116.204 t}+(0.6\, +0.8 i)}, \end{aligned}$$

87 $$\begin{aligned} \Upsilon (10, t)= & {} \frac{1}{(0.0322582\, +0.00335672 i) e^{116.205 t}+(\text {4.336808689942018}*{}^{\wedge }\text {-19}-0.0324324 i) e^{0.00045 t}}. \end{aligned}$$

## Conclusion

This article, is deals the numerical and analytical study for the stochastic Burgers’ equation under the influence of time noise. The stochastic Burgers’ equation plays an important role in the fields of applied mathematics such as fluid dynamics, gas dynamics, traffic flow, and nonlinear acoustics. The existence of result is successfully shown by the help of Schauder fixed point theorem. The length of contraction mapping this define under the condition $\rho$ which represents the length of mapping where at least one solution is exist. For the numerical results the proposed stochastic finite difference scheme developed. The analysis of proposed scheme under the multiplicative time noise is visualized like consistency of the scheme and linearize analysis as well. The consistency is check under the mean square sense while the stability condition is gained by the help of Von-Neumann criteria. Meanwhile, the stochastic exact solutions are constructed successfully by using the generalized exponential rational function method. These stochastic exact solitary wave solutions are obtained in the form of hyperbolic, trigonometric and exponential functions. Mainly, in this study we focused on the comparisons of numerical result with stochastic exact solitary wave solutions. The ICs and BCs are required to compute the numerical results that are are constructed from the newly constructed solitary wave solutions. Some stochastic ESW solutions chosen and compare them with proposed stochastic finite difference scheme they will provided us the similar behavior and the effect of the randomness is also clearly visualized from the plots. The different 3D, 2D and corresponding contours are drawn for the different values of $\sigma$. The 3D and line plots are dispatched that are shown the similar behavior by choosing the different values of parameters. These results are the main innovation of this study under the noise effects. This study is very helpful in the future direction for the researchers to apply the techniques on the stochastic NLPDEs. Moreover the comparison of the results is a new direction in the modern era of research.

## Data Availability

All data generated or analysed during this study are included in this article.

## References

[1] Chambers, D. H., Adrian, R. J., Moin, P., Stewart, D. S. & Sung, H. J. Karhunen–Loéve expansion of Burgers’ model of turbulence. *Phys. Fluids* **31**(9), 2573–2582 (1988).

[2] Baber, M. Z. *et al.* Comparative analysis of numerical with optical soliton solutions of stochastic Gross–Pitaevskii equation in dispersive media. *Results Phys.* **44**, 106175 (2023).

[3] Baber, M. Z., Seadway, A. R., Ahmed, N., Iqbal, M. S. & Yasin, M. W. Selection of solitons coinciding the numerical solutions for uniquely solvable physical problems: A comparative study for the nonlinear stochastic Gross–Pitaevskii equation in dispersive media. *Int. J. Mod. Phys. B* **37**, 2350191 (2022).

[4] Da Prato, G. & Da Prato, G. The stochastic Burgers equation. In *Kolmogorov Equations for Stochastic PDEs* (ed. Da Prato, G.) 131–153 (Springer, 2004).

[5] Iqbal, M. S. *et al.* Numerical simulations of nonlinear stochastic Newell–Whitehead–Segel equation and its measurable properties. *J. Comput. Appl. Math.* **418**, 114618 (2023).

[6] Arif, M. S., Abodayeh, K. & Nawaz, Y. A reliable computational scheme for stochastic reaction–diffusion nonlinear chemical model. *Axioms* **12**(5), 460 (2023).

[7] Raza, A., Arif, M. S. & Rafiq, M. A reliable numerical analysis for stochastic dengue epidemic model with incubation period of virus. *Adv. Differ. Equ.* **2019**(1), 1–19 (2019).

[8] Kovács, M., Larsson, S. & Lindgren, F. On the backward Euler approximation of the stochastic Allen–Cahn equation. *J. Appl. Probab.* **52**(2), 323–338 (2015).

[9] Yasin, M. W. *et al.* Numerical scheme and analytical solutions to the stochastic nonlinear advection diffusion dynamical model. *Int. J. Nonlinear Sci. Numer. Simul.* **24**, 467 (2021).

[10] Yasin, M. W. *et al.* Reliable numerical analysis for stochastic reaction–diffusion system. *Phys. Scr.* **98**(1), 015209 (2022).

[11] Yasin, M. W. *et al.* Spatio-temporal numerical modeling of stochastic predator–prey model. *Sci. Rep.* **13**, 1990 (2023). 36737648 10.1038/s41598-023-28324-6 PMC10439196

[12] Washenberger, M. J. *et al.* Soliton solutions of fractional stochastic Kraenkel–Manna–Merle equations in ferromagnetic materials. *Fractal Fract.* **7**(4), 328 (2023).

[13] Mohammed, W. W., Al-Askar, F. M., Cesarano, C. & El-Morshedy, M. Solitary wave solution of a generalized fractional-stochastic nonlinear wave equation for a liquid with gas bubbles. *Mathematics* **11**(7), 1692 (2023).

[14] Mohammed, W. W., Al-Askar, F. M., Cesarano, C. & Aly, E. S. The soliton solutions of the stochastic shallow water wave equations in the sense of beta-derivative. *Mathematics* **11**(6), 1338 (2023).

[15] Mohammed, W. W., Al-Askar, F. M., Cesarano, C. & El-Morshedy, M. Solitary wave solutions of the fractional-stochastic quantum Zakharov–Kuznetsov equation arises in quantum magneto plasma. *Mathematics* **11**(2), 488 (2023).

[16] Albosaily, S., Mohammed, W. & El-Morshedy, M. The exact solutions of the fractional-stochastic Fokas–Lenells equation in optical fiber communication. *Electron. Res. Arch.* **31**(6), 3552–3567 (2023).

[17] Shaikh, T. S. *et al.* Investigation of solitary wave structures for the stochastic Nizhnik–Novikov–Veselov (SNNV) system. *Results Phys.* **48**, 106389 (2023).

[18] Shaikh, T. S. *et al.* On the soliton solutions for the stochastic Konno–Oono system in magnetic field with the presence of noise. *Mathematics* **11**(6), 1472 (2023).

[19] Assiri, T. A., Saifullah, S., Khan, M. A. & Sun, M. Some new optical solitary waves solutions of a third order dispersive Schrödinger equation with Kerr nonlinearity using an efficient approach associated with Riccati equation. *Opt. Quant. Electron.* **56**(4), 1–13 (2024).

[20] Khan, A., Saifullah, S., Ahmad, S., Khan, M. A. & Rahman, M. U. Dynamical properties and new optical soliton solutions of a generalized nonlinear Schrödinger equation. *Eur. Phys. J. Plus* **138**(11), 1059 (2023).

[21] Ali, A., Ahmad, J. & Javed, S. Solitary wave solutions for the originating waves that propagate of the fractional Wazwaz–Benjamin–Bona–Mahony system. *Alex. Eng. J.* **69**, 121–133 (2023).

[22] Chahlaoui, Y., Ali, A. & Javed, S. Study the behavior of soliton solution, modulation instability and sensitive analysis to fractional nonlinear Schrödinger model with Kerr Law nonlinearity. *Ain Shams Eng. J.* **15**(3), 102567 (2024).

[23] Ali, A., Ahmad, J. & Javed, S. Exact soliton solutions and stability analysis to (3 + 1)-dimensional nonlinear Schrödinger model. *Alex. Eng. J.* **76**, 747–756 (2023).

[24] Gonçalves, P., Jara, M. & Sethuraman, S. *A Stochastic Burgers Equation from a Class of Microscopic Interactions* (2015).

[25] Al-Askar, F. M., Mohammed, W. W. & El-Morshedy, M. The analytical solutions for stochastic fractional-space Burgers’ equation. *J. Math.* **2022**, 1–8 (2022).

[26] Mohammed, W. W., Albosaily, S., Iqbal, N. & El-Morshedy, M. The effect of multiplicative noise on the exact solutions of the stochastic Burgers’ equation. In *Waves in Random and Complex Media* (eds Mohammed, W. W. *et al.*) 1–13 (Taylor & Francis, 2021).

[27] Blomker, D. & Jentzen, A. Galerkin approximations for the stochastic Burgers equation. *SIAM J. Numer. Anal.* **51**(1), 694–715 (2013).

[28] Kutluay, S. E. L. Ç. U. K., Bahadir, A. R. & Özdes, A. Numerical solution of one-dimensional Burgers equation: Explicit and exact-explicit finite difference methods. *J. Comput. Appl. Math.* **103**(2), 251–261 (1999).

[29] Xie, S. S., Heo, S., Kim, S., Woo, G. & Yi, S. Numerical solution of one-dimensional Burgers’ equation using reproducing kernel function. *J. Comput. Appl. Math.* **214**(2), 417–434 (2008).

[30] Mohammed, W. W., Iqbal, N., Ali, A. & El-Morshedy, M. Exact solutions of the stochastic new coupled Konno–Oono equation. *Results Phys.* **21**, 103830 (2021).

[31] Iqbal, M. S. *Solutions of Boundary Value Problems for Nonlinear Partial Differential Equations by Fixed Point Methods* (2011).

[32] Baber, M. Z. *et al.* Comparative analysis of numerical and newly constructed soliton solutions of stochastic Fisher-type equations in a sufficiently long habitat. *Int. J. Mod. Phys. B* **37**, 2350155 (2022).

[33] Yasin, M. W. *et al.* Numerical scheme and stability analysis of stochastic Fitzhugh–Nagumo model. *Results Phys.* **32**, 105023 (2022).

[34] Wang, C., Qiu, Z. & Wu, D. Numerical analysis of uncertain temperature field by stochastic finite difference method. *Sci. China Phys. Mech. Astron.* **57**, 698–707 (2014).

[35] Mohammed, W. W., Ahmad, H., Boulares, H., Khelifi, F. & El-Morshedy, M. Exact solutions of Hirota–Maccari system forced by multiplicative noise in the Itô sense. *J. Low Freq. Noise Vib. Act. Control* **41**(1), 74–84 (2022).

[36] Mohammed, W. W. & El-Morshedy, M. The influence of multiplicative noise on the stochastic exact solutions of the Nizhnik–Novikov–Veselov system. *Math. Comput. Simul.* **190**, 192–202 (2021).

[37] Iqbal, M. S., Seadawy, A. R., Baber, M. Z. & Qasim, M. Application of modified exponential rational function method to Jaulent–Miodek system leading to exact classical solutions. *Chaos Solitons Fractals* **164**, 112600 (2022).

[38] Ghanbari, B., Osman, M. S. & Baleanu, D. Generalized exponential rational function method for extended Zakharov–Kuzetsov equation with conformable derivative. *Mod. Phys. Lett. A* **34**(20), 1950155 (2019).

